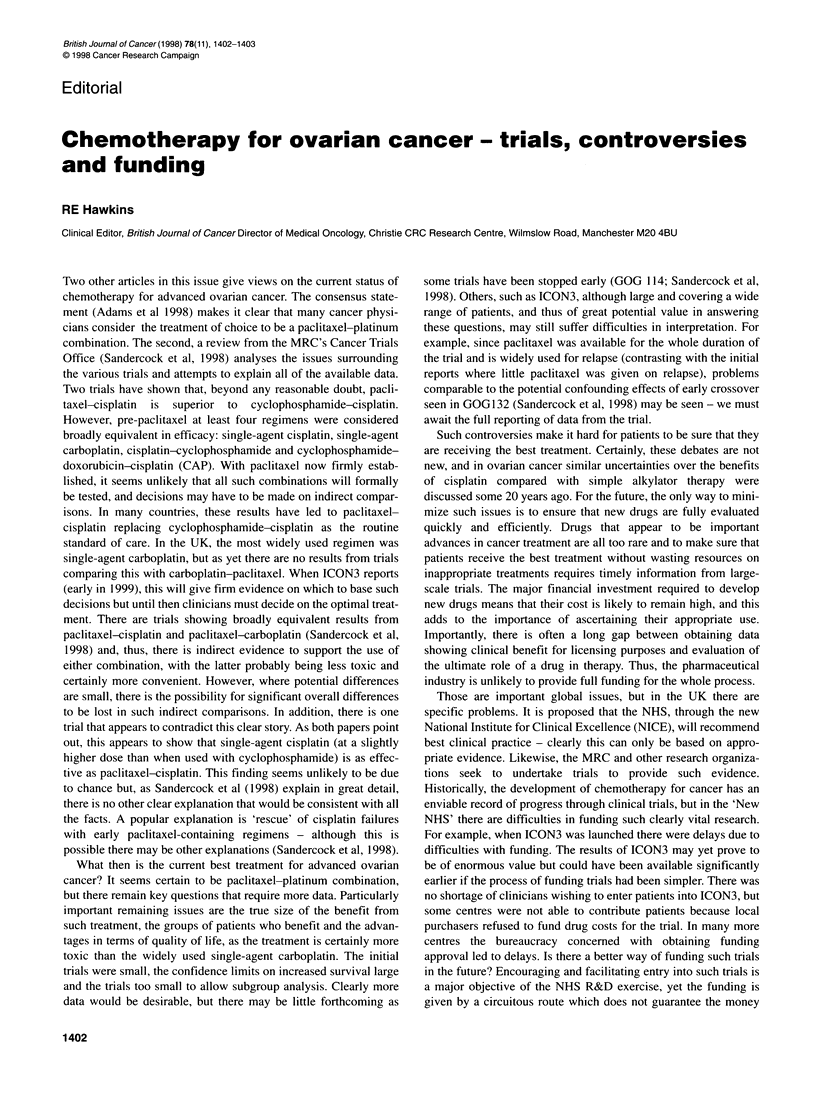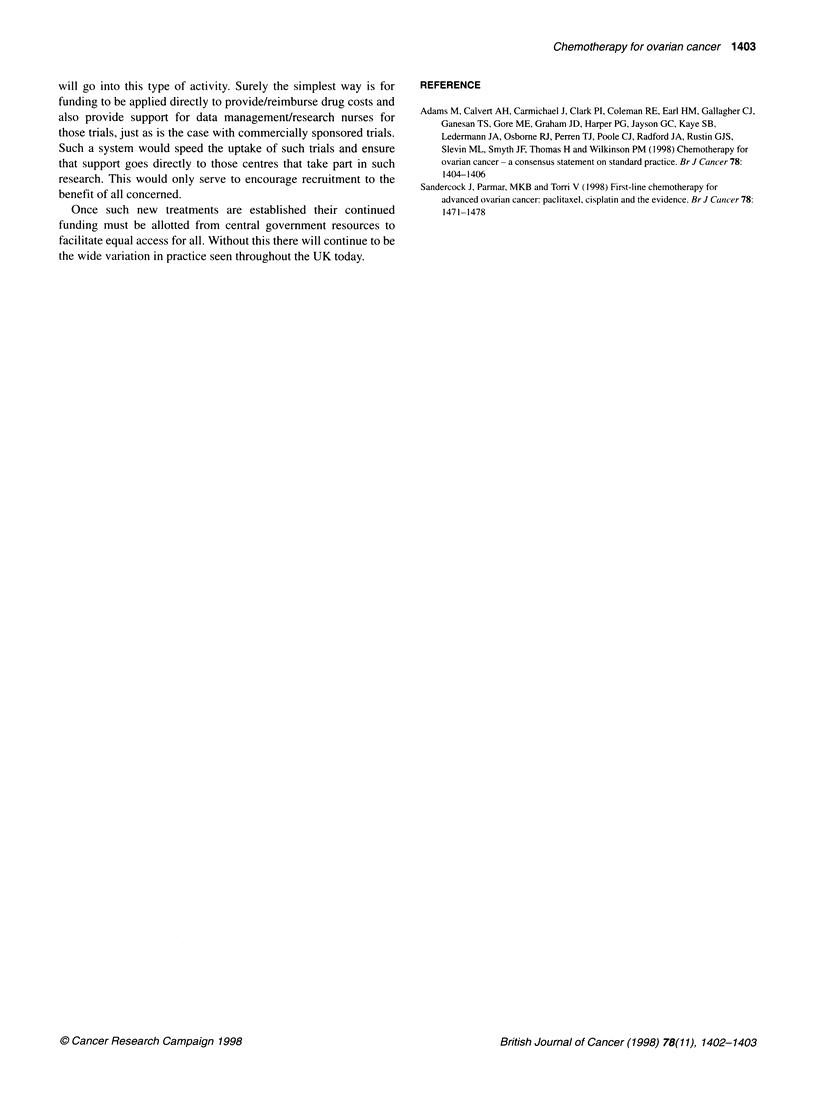# Chemotherapy for ovarian cancer--trials, controversies and funding.

**DOI:** 10.1038/bjc.1998.698

**Published:** 1998-12

**Authors:** R. E. Hawkins


British Journal of Cancer (1998) 78(11), 1402–1403
© 1998 Cancer Research Campaign

Editorial

### Chemotherapy for ovarian cancer – trials, controversies and funding

**RE Hawkins**

Clinical Editor, *British Journal of Cancer* Director of Medical Oncology, Christie CRC Research Centre, Wilmslow Road, Manchester M20 4BU

Two other articles in this issue give views on the current status of chemotherapy for advanced ovarian cancer. The consensus statement (Adams et al 1998) makes it clear that many cancer physicians consider the treatment of choice to be a paclitaxel–platinum combination. The second, a review from the MRC's Cancer Trials Office (Sandercock et al, 1998) analyses the issues surrounding the various trials and attempts to explain all of the available data. Two trials have shown that, beyond any reasonable doubt, paclitaxel–cisplatin is superior to cyclophosphamide–cisplatin. However, pre-paclitaxel at least four regimens were considered broadly equivalent in efficacy: single-agent cisplatin, single-agent carboplatin, cisplatin–cyclophosphamide and cyclophosphamide–doxorubicin–cisplatin (CAP). With paclitaxel now firmly established, it seems unlikely that all such combinations will formally be tested, and decisions may have to be made on indirect comparisons. In many countries, these results have led to paclitaxel–cisplatin replacing cyclophosphamide–cisplatin as the routine standard of care. In the UK, the most widely used regimen was single-agent carboplatin, but as yet there are no results from trials comparing this with carboplatin–paclitaxel. When ICON3 reports (early in 1999), this will give firm evidence on which to base such decisions but until then clinicians must decide on the optimal treatment. There are trials showing broadly equivalent results from paclitaxel–cisplatin and paclitaxel–carboplatin (Sandercock et al, 1998) and, thus, there is indirect evidence to support the use of either combination, with the latter probably being less toxic and certainly more convenient. However, where potential differences are small, there is the possibility for significant overall differences to be lost in such indirect comparisons. In addition, there is one trial that appears to contradict this clear story. As both papers point out, this appears to show that single-agent cisplatin (at a slightly higher dose than when used with cyclophosphamide) is as effective as paclitaxel–cisplatin. This finding seems unlikely to be due to chance but, as Sandercock et al (1998) explain in great detail, there is no other clear explanation that would be consistent with all the facts. A popular explanation is 'rescue' of cisplatin failures with early paclitaxel-containing regimens – although this is possible there may be other explanations (Sandercock et al, 1998).

What then is the current best treatment for advanced ovarian cancer? It seems certain to be paclitaxel–platinum combination, but there remain key questions that require more data. Particularly important remaining issues are the true size of the benefit from such treatment, the groups of patients who benefit and the advantages in terms of quality of life, as the treatment is certainly more toxic than the widely used single-agent carboplatin. The initial trials were small, the confidence limits on increased survival large and the trials too small to allow subgroup analysis. Clearly more data would be desirable, but there may be little forthcoming as some trials have been stopped early (GOG 114; Sandercock et al, 1998). Others, such as ICON3, although large and covering a wide range of patients, and thus of great potential value in answering these questions, may still suffer difficulties in interpretation. For example, since paclitaxel was available for the whole duration of the trial and is widely used for relapse (contrasting with the initial reports where little paclitaxel was given on relapse), problems comparable to the potential confounding effects of early crossover seen in GOG132 (Sandercock et al, 1998) may be seen – we must await the full reporting of data from the trial.

Such controversies make it hard for patients to be sure that they are receiving the best treatment. Certainly, these debates are not new, and in ovarian cancer similar uncertainties over the benefits of cisplatin compared with simple alkylator therapy were discussed some 20 years ago. For the future, the only way to minimize such issues is to ensure that new drugs are fully evaluated quickly and efficiently. Drugs that appear to be important advances in cancer treatment are all too rare and to make sure that patients receive the best treatment without wasting resources on inappropriate treatments requires timely information from large-scale trials. The major financial investment required to develop new drugs means that their cost is likely to remain high, and this adds to the importance of ascertaining their appropriate use. Importantly, there is often a long gap between obtaining data showing clinical benefit for licensing purposes and evaluation of the ultimate role of a drug in therapy. Thus, the pharmaceutical industry is unlikely to provide full funding for the whole process.

Those are important global issues, but in the UK there are specific problems. It is proposed that the NHS, through the new National Institute for Clinical Excellence (NICE), will recommend best clinical practice – clearly this can only be based on appropriate evidence. Likewise, the MRC and other research organizations seek to undertake trials to provide such evidence. Historically, the development of chemotherapy for cancer has an enviable record of progress through clinical trials, but in the 'New NHS' there are difficulties in funding such clearly vital research. For example, when ICON3 was launched there were delays due to difficulties with funding. The results of ICON3 may yet prove to be of enormous value but could have been available significantly earlier if the process of funding trials had been simpler. There was no shortage of clinicians wishing to enter patients into ICON3, but some centres were not able to contribute patients because local purchasers refused to fund drug costs for the trial. In many more centres the bureaucracy concerned with obtaining funding approval led to delays. Is there a better way of funding such trials in the future? Encouraging and facilitating entry into such trials is a major objective of the NHS R&D exercise, yet the funding is given by a circuitous route which does not guarantee the money will go into this type of activity. Surely the simplest way is for funding to be applied directly to provide/reimburse drug costs and also provide support for data management/research nurses for those trials, just as is the case with commercially sponsored trials. Such a system would speed the uptake of such trials and ensure that support goes directly to those centres that take part in such research. This would only serve to encourage recruitment to the benefit of all concerned.

Once such new treatments are established their continued funding must be allotted from central government resources to facilitate equal access for all. Without this there will continue to be the wide variation in practice seen throughout the UK today.

### REFERENCE

Adams M, Calvert AH, Carmichael J, Clark PI, Coleman RE, Earl HM, Gallagher CJ, Ganesan TS, Gore ME, Graham JD, Harper PG, Jayson GC, Kaye SB, Ledermann JA, Osborne RJ, Perren TJ, Poole CJ, Radford JA, Rustin GJS, Slevin ML, Smyth JF, Thomas H and Wilkinson PM (1998) Chemotherapy for ovarian cancer – a consensus statement on standard practice. *Br J Cancer* **78**: 1404–1406

Sandercock J, Parmar, MKB and Torri V (1998) First-line chemotherapy for advanced ovarian cancer: paclitaxel, cisplatin and the evidence. *Br J Cancer* **78**: 1471–1478

© Cancer Research Campaign 1998